# Analysis of nationwide availability of negotiated drugs in China’s National Reimbursement Drug List

**DOI:** 10.3389/fpubh.2025.1718627

**Published:** 2026-01-08

**Authors:** Yumeng Zhang, Wenqi Zhang, Jizuoni Deng, Yuhan Liu, Lihua Sun

**Affiliations:** 1 Department of Pharmacy Administration, School of Business Administration, Shenyang Pharmaceutical University, Shenyang, Liaoning, China; 2 Shanghai Health Development Research Center (Shanghai Medical Information Center), Shanghai, China

**Keywords:** allocation, availability, designated retail pharmacies, healthcare institutions, national negotiated drugs

## Abstract

**Objective:**

This study aims to evaluate the availability of Price-Negotiated Drugs under China’s National Reimbursement Drug List in healthcare institutions and designated retail pharmacies.

**Methods:**

This cross-sectional study uses data from the National Health Insurance Service Platform (extracted in September 2025) to evaluate the availability of 383 National Negotiated Drugs in healthcare institutions and retail pharmacies nationwide. According to the World Health Organization (WHO)/Health Action International (HAI) framework, availability was assessed based on the availability rate and allocation rate. Spearman’s rank correlation test (*α* = 0.05) was applied to examine associations between drug availability and factors including the timing of formulary inclusion, per capita disposable income, regional gross domestic product (GDP), the number of relevant policy releases and Pharmacists, the accumulated balance of health insurance funds, and the average annual number of visits per person.

**Results:**

The overall availability of negotiated drugs was low, with mean availability rates of 2.08% in healthcare institutions and 0.13% in designated retail pharmacies. Although drugs were predominantly concentrated in tertiary hospitals, their average availability there remained modest at 9.84%. Substantial provincial disparities were observed in the allocation of tertiary hospitals, with the eastern regions showing relatively higher rates. Drugs added to the formulary earlier and those with higher clinical demand were more available. Spearman’s correlation analysis indicated significant associations between drug availability and timing of formulary inclusion (*r* = −0.53, *p* < 0.001), per capita disposable income (*r* = 0.45, *p* = 0.012), regional GDP (*r* = 0.49, *p* = 0.005), the number of relevant policy releases (*r* = 0.57, *p* < 0.001), the number of Pharmacists (*r* = 0.43, *p* = 0.017), the accumulated balance of health insurance funds (*r* = 0.61, *p* < 0.001), and the average annual number of visits per person (*r* = 0.62, *p* < 0.001).

**Conclusion:**

According to the WHO/HAI standards, the overall availability of negotiated drugs in China remains relatively low, with notable institutional stratification and regional disparities. This finding suggests that policy efforts should focus on strengthening supply incentives and promoting rational drug use. Additionally, policymakers should consider the differences across regions and institutions when advancing drug availability to ensure equitable access to medicines.

## 1 Introduction

Against the backdrop of deepening healthcare security reform, Price-Negotiated Drugs under China’s National Reimbursement Drug List (hereinafter, “negotiated drugs”) have emerged as one of the most significant policy innovations in China’s drug reimbursement system in recent years. This initiative is designed to reduce patients’ financial burden, particularly for those suffering from major, chronic, and severe illnesses (1). Beyond fulfilling basic health needs, the program is regarded as a critical measure to enhance the efficiency of medical insurance fund utilization and promote the high-quality development of the pharmaceutical industry.

Since 2016, mechanisms for the inclusion and management of negotiated drugs have been gradually established and refined. In 2017, the first negotiated drugs were incorporated into the reimbursement list. With the establishment of the National Healthcare Security Administration (NHSA) in 2018, the responsibility for drug negotiations was centralized, enabling the institutionalization of a dynamic adjustment mechanism for the reimbursement formulary (2, 3). Approximately 80% of new drugs now enter the reimbursement list within 2 years of market approval, and some innovative drugs are included in the very year of their launch (4). While this trend has significantly improved drug availability, its long-term value and necessity remain a matter of debate. Concerns persist regarding drugs approved with limited clinical evidence or with readily available alternatives, raising questions about whether rapid inclusion in the reimbursement list is always warranted. In other words, the expansion of the formulary does not necessarily guarantee that patients derive meaningful and equitable benefits (5).

Moreover, in practice, the inclusion of innovative drugs through national negotiations merely marks the beginning of ensuring patient access to these drugs, rather than the endpoint. While the NHSA has issued policies explicitly prohibiting restrictions on the allocation and use of negotiated drugs due to factors such as global budget control, formulary limits, and drug expenditure ratios, many performance indicators within its evaluation framework still fail to exclude the impact of negotiated drugs. These include indicators like medical revenue share, the proportion of essential drugs, and average treatment costs (6–11). According to a survey conducted by China Hospital President magazine, policy-related factors such as global budget control and essential drug share assessments are significant constraints on hospital decision-making, with an influence level exceeding 60% (12). To address these hospital entry barriers, the NHSA, together with the National Health Commission, has successively issued the *Guiding Opinions on Establishing and Improving the Dual-Channel Management Mechanism for Negotiated Drugs* (13) and the *Notice on Ensuring the Sustainable Implementation of Negotiated Drugs under Normalized Mechanisms* (14). These measures have expanded the supply channels of negotiated drugs to designated retail pharmacies, which, alongside designated healthcare institutions, jointly promote their adoption and rational use. Currently, conducting in-depth and systematic research on the real-world implementation of these policies is of utmost importance. Such work is critical not only for optimizing the management framework of negotiated drugs and enhancing the effectiveness of policy execution but also for providing robust evidence to support the ongoing refinement of negotiation policies, with significant practical and long-term implications.

However, existing research primarily focuses on macro-policy discussions or is confined to case studies of specific regions (15) or individual healthcare institutions (16, 17). While these studies provide valuable perspectives, their limitations lie in being restricted to a single region or healthcare institution. They lack systematic, nationwide analyses across different regions and healthcare tiers (18). This restricts our comprehensive understanding of the practical supply and regional differences in the adoption of negotiated drugs, as well as weakens the evidence base for policy dissemination and refinement. More importantly, existing research has not yet delved deeply into the potential factors related to drug availability, which may result in a limited understanding of the availability of nationally negotiated drugs across regions and may fail to fully capture the actual situation.

In light of this, the present study makes a novel contribution by conducting a systematic nationwide analysis that includes primary, secondary, and tertiary hospitals as well as designated retail pharmacies. It provides a comprehensive assessment of the current availability of nationally negotiated drugs and the disparities in their implementation across different regions and healthcare institution levels. Furthermore, to address the gap in understanding how inter-provincial differences impact drug availability, this study employs Spearman’s rank correlation analysis to evaluate the relationship between key factors—such as regional economic development, government policy responsiveness, pharmaceutical management capacity in healthcare institutions, the medical security capacity, and residents’ healthcare service demand—and the availability of nationally negotiated drugs across provinces. This analysis not only identifies key factors related to drug availability but also provides important empirical evidence for understanding the structural differences in the implementation of nationally negotiated drugs in various regions. The study aims to provide a more comprehensive and reliable empirical reference for optimizing the implementation of nationally negotiated drugs and offer policymakers more practical and actionable guidance for decision-making.

## 2 Data and methods

### 2.1 Data sources

The core cross-sectional data for this study were obtained from the National Health Insurance Service Platform, which systematically records the provision of negotiated drugs included in the National Reimbursement Drug List (NRDL) across designated healthcare institutions and retail pharmacies nationwide. In total, the platform covers 404 negotiated drugs that remain within the valid agreement period. To focus more precisely on the actual provision of newly negotiated drugs in both designated healthcare institutions and retail pharmacies, and to reflect the willingness or capacity of these institutions and pharmacies to supply these drugs, the following selection criteria were applied to the sample drugs:

(1) Drugs that were initially part of the conventional NRDL and later transferred to the negotiated category were excluded.
(2) Drugs that, at the time of data extraction (September 2025), had not been supplied in any designated institution due to company-related reasons were also excluded.

After applying these criteria, 383 negotiated drugs remained as the final analytic sample. For each drug, we extracted the names of healthcare institutions and designated retail pharmacies where the drugs were available, and further queried the tier of each institution in the Designated Medical Institutions Query section of the National Health Insurance Service Platform. This information was used to construct the dataset for this study. To ensure the accuracy and consistency of the data, rigorous measures were taken during the data cleaning process. Three independent researchers conducted the data cleaning and cross-checked the final results. Through this process, we ensured that potential errors were identified and corrected, thereby minimizing human errors and enhancing the reliability of the data. Additional data, including disease area classifications of the drugs, the year of their initial inclusion in the NRDL, the number of medical institutions at national and provincial levels, per capita disposable income, regional gross domestic product (GDP), and relevant provincial (municipal) policy documents, the number of Pharmacists, the accumulated balance of health insurance funds, and the average annual number of visits per person, were collected from publicly available sources such as yaozhi Network, Menet, the National Bureau of Statistics, and the official websites of provincial (municipal) healthcare security administrations. For policy data, we applied a standardized inclusion criterion to ensure that only substantive documents were counted, thereby guaranteeing that cross-provincial comparisons of policy volume were meaningful and comparable (specific policies selected are detailed in Supplementary Table S1).

### 2.2 Indicator design

Following the methodological framework of the World Health Organization (WHO)/Health Action International (HAI), this study systematically evaluated the implementation of negotiated drugs from two perspectives: availability rate and allocation rate (19, 20). The availability rate reflects the proportion of institutions (or regions) in which a given negotiated drug is available, indicating its distribution across institutions or regions. The allocation rate reflects the proportion of negotiated drugs available in a specific healthcare institution (or region), highlighting the extent of negotiated drug availability in that institution or region. According to WHO/HAI standards (21): Absent (0%): the drug was not available at any institutions. Very low (<30%): the drug was rarely available. Low (30–49%): the drug was available in some institutions, but was limited. Relatively high (50–80%): the drug was available in many institutions. High (>80%): the drug was widely available.

The specific measurement indicators are defined as follows:

(1) Availability Rate

$$\begin{array}{l} \text{Availability rate of}\;a\;\text{given drug in institutions}\;(\text{or regions})=\\ \frac{\begin{array}{l} \text{Number of institutions}\;(\text{or regions})\\ \text{where the}\;me\text{dicine is available} \end{array}}{\text{Total number of institutions}\;(\text{or regions})}\times 100\% \end{array}$$

(2) Allocation Rate

$$\begin{array}{l} \text{Allocation rate of drugs in institutions}\;\;(\text{or regions})=\\ \frac{\text{Number of negotiated}\;\text{drug}s\;\text{available}}{\text{Total number of sample}\;\text{drugs}}\times 100\% \end{array}$$

$$\begin{array}{l} \text{Average allocation rate of institutions}\;(\text{or regions})=\\ \frac{\begin{array}{l} \text{Average number of negotiated drugs}\;\\ \text{available in institutions}\;(\text{or regions}) \end{array}}{\text{Total number of sample}\;\text{drug}s}\times 100\% \end{array}$$

### 2.3 Statistical analysis

This study employed the following statistical methods for descriptive analyses and factor correlation assessments:

#### 2.3.1 Descriptive analysis

First, the availability of negotiated drugs was systematically described across different tiers of healthcare institutions (primary, secondary, and tertiary hospitals) as well as designated retail pharmacies. Second, cross-provincial comparisons of allocation levels were conducted to reveal regional disparities. To enhance interpretability and clarity, bar charts, heat maps, and other visualization techniques were used to present availability rates and regional distribution patterns, thereby providing a more comprehensive picture of availability differences across healthcare systems and geographic regions.

#### 2.3.2 Correlation analysis

To further explore the potential impact of provincial differences on the availability of NRDL-negotiated drugs, this study examines a range of influencing factors, including the timing of drug inclusion, regional economic development, government policy responsiveness, pharmaceutical management capabilities in healthcare institutions, the medical security capacity, and the demand for residents’ healthcare services. Specifically, regional GDP and per capita disposable income were chosen as core indicators to measure the economic level of each province, as these indicators provide a comprehensive reflection of the region’s economic development and are representative of economic factors (22, 23). Therefore, this study uses these two variables as the primary tools for assessing economic development levels.

Pharmacists play a critical role in drug management, distribution, procurement, and inventory management. Existing research has shown that increasing the number of pharmacists contributes to improving drug availability and the quality of drug supply (24). Therefore, this study incorporates the number of pharmacists into the analytical framework as a variable to measure the drug management capacity of healthcare institutions in different regions. The accumulated balance of health insurance funds is an important variable for measuring regional medical security capacity, reflecting the financial status and payment capability of the health insurance fund. A well-functioning medical security system is crucial for drug distribution and availability. In addition, previous studies have pointed out that the number of visits per person per year is a key variable for measuring healthcare service demand (25). Consequently, this study introduces the “average annual number of visits per person” as a variable to reflect actual patient demand and further analyze its impact on drug availability.

Based on the aforementioned indicators, this study employs Spearman’s rank correlation coefficient to analyze the correlation between drug availability and the following variables: the time of inclusion in the medical insurance catalog, regional economic development level (GDP and per capita disposable income), number of policy documents, number of pharmacists, accumulated balance of health insurance funds, and average annual visits per person. A significance level of *α* = 0.05 was set. The goal of this analysis is to reveal the potential impact of these variables on drug availability and provide empirical evidence for policymaking.

All data processing and statistical analyses were conducted using Excel 2021, SPSS 26.0, R 4.4.1, and RStudio 2024.04.2. As the study relied exclusively on publicly available data and did not involve individual patient-level information, ethical review was not required.

## 3 Results

### 3.1 Overall availability in healthcare institutions and retail pharmacies

**Figure 1:**
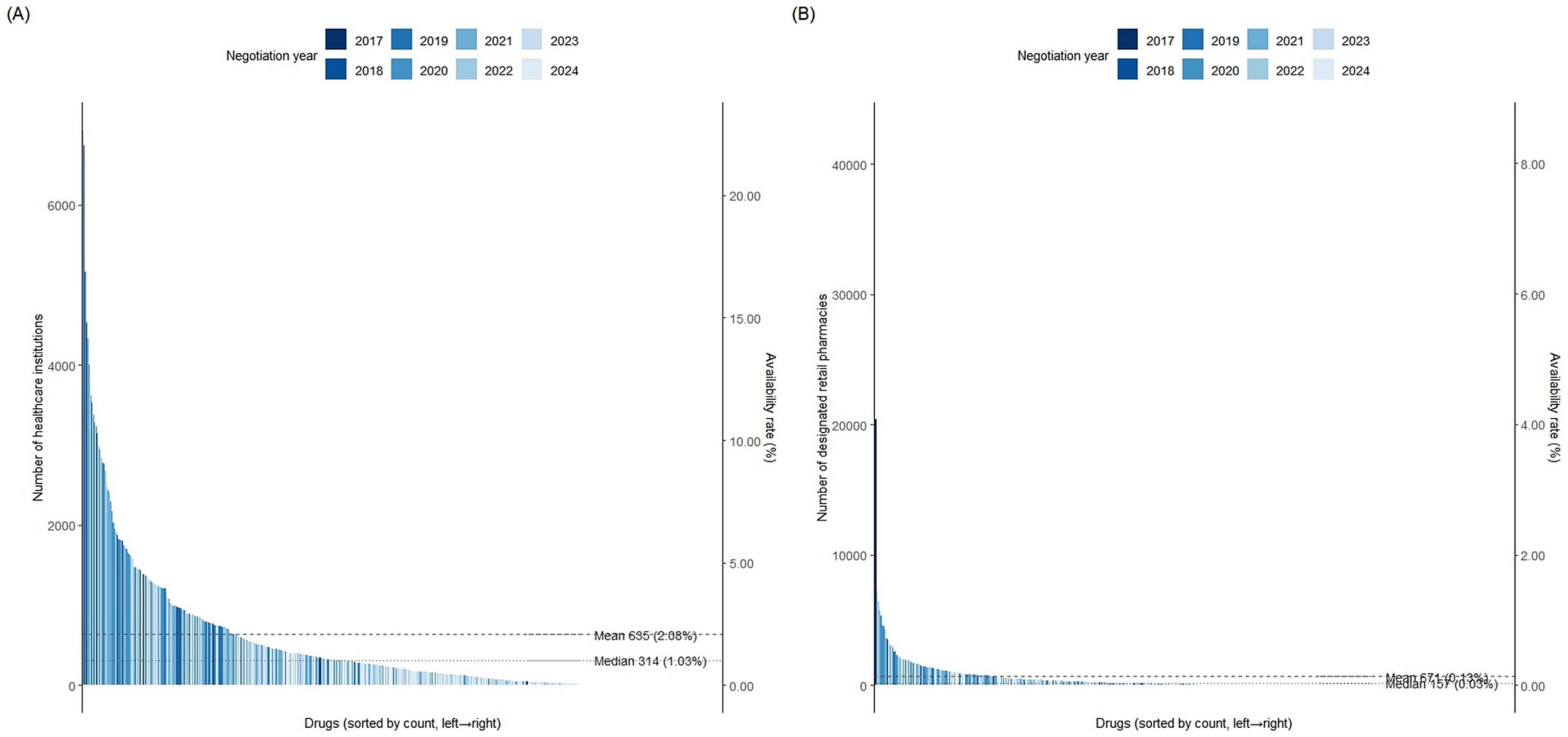
Frequency distribution and average availability rates of negotiated drugs in healthcare institutions **(A)** and retail pharmacies **(B)**.

Overall, the average availability of negotiated drugs remained low in both designated healthcare institutions and retail pharmacies, at 2.08 and 0.13%, respectively (Figure 1). Stratified analysis further showed that the average availability rates in primary, secondary, and tertiary hospitals were 0.16, 1.58 and 9.64%, respectively (Supplementary Table S2). These findings indicate that tertiary hospitals serve as the primary sites for providing negotiated drugs and remain the main access point for patients. Consequently, this study considers the availability rate in tertiary hospitals as a key indicator for evaluating the institutional adoption of negotiated drugs.

**Figure 2:**
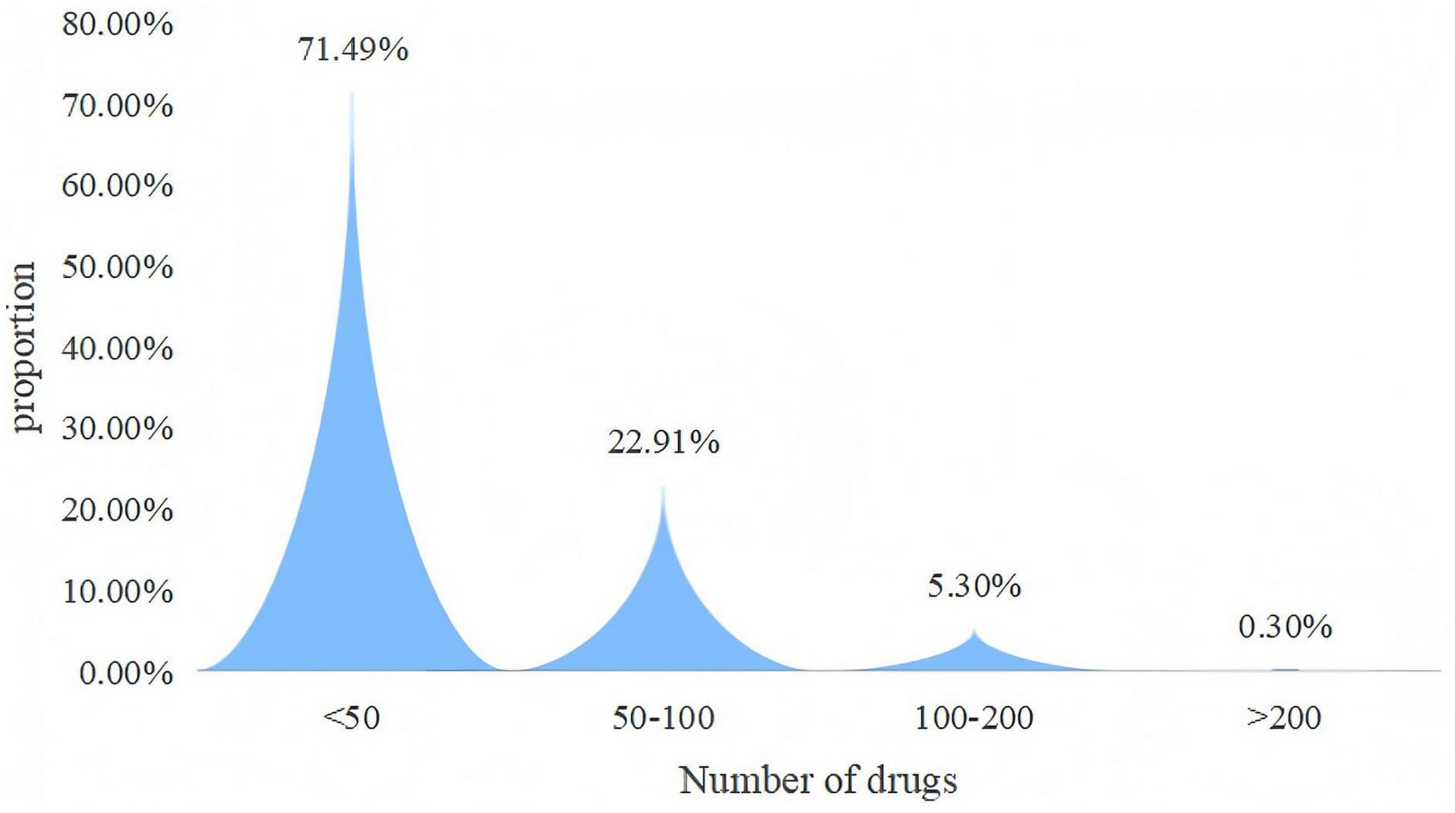
Proportional distribution of tertiary hospitals by number of negotiated drugs supplied (<50, 50–100, 100–200, >200).

**Figure 3:**
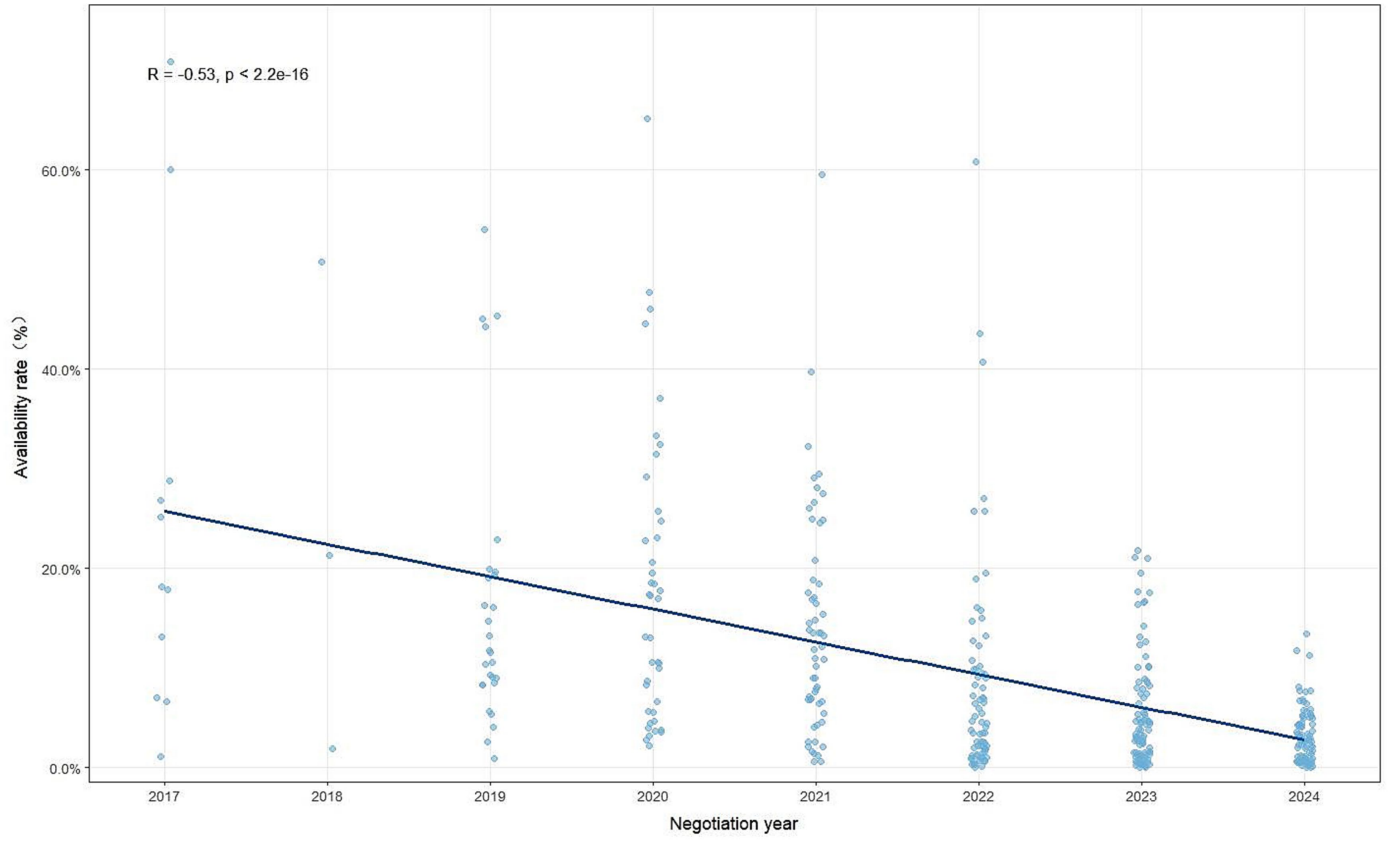
Correlation between negotiation year and availability rate in tertiary hospitals.

Within tertiary hospitals, overall availability was still limited. Of the 383 sampled drugs, only 21 (5.48%) had an availability rate exceeding 30% (Table 1), and merely 0.3% of tertiary hospitals supplied more than 200 negotiated drugs (Figure 2). By drug category, the average availability rate of Western medicines in tertiary hospitals was 10.12%, while that of Traditional Chinese Medicine (TCM) was 6.41%. Among them, the availability rate of Xuebijing injection was relatively high (44.25%), while the availability rates of most other traditional Chinese medicines were generally low (Supplementary Table S3). Apparent differences were observed across negotiation years: drugs negotiated in 2017 had an average availability rate of 25.07%, compared with only 3.32% for those negotiated in 2024 (Table 2). Spearman’s correlation analysis confirmed this pattern, revealing a significant negative correlation between availability rate and negotiation year (*R* = −0.53, *p* < 0.001), suggesting a declining trend in availability with more recent negotiations (Figure 3).

**Table 1:** Number of negotiated drugs by availability-rate category in tertiary hospitals.

Availability rate (%)	Number of drugs
<30	362
30–50	14
50–80	7
>80	0

**Table 2:** Average availability rates of negotiated drugs in tertiary hospitals by negotiation years.

Negotiation year	2017	2018	2019	2020	2021	2022	2023	2024
Average availability rate (%)	25.07	24.66	16.60	18.15	13.96	8.05	5.50	3.32

In designated retail pharmacies, 321 negotiated drugs were identified, all of which were also available through hospital channels. This finding suggests that hospitals continue to be the primary channel for negotiated drugs. In contrast, 62 drugs were supplied exclusively by hospitals and not distributed through retail pharmacies, 64.52% of which were newly added to the negotiation list in 2023–2024 (Supplementary Table S4), and among the drugs that had entered retail pharmacies, oral and topical formulations exhibited relatively higher availability rates (Table 3).

**Table 3:** Average availability rates of different formulations in designated retail pharmacies.

Formulation	Average availability rate (%)
Inhalation	0.07
Injection	0.12
Other formulations	0.01
Oral	0.15
Topical	0.25

To compare differences between the two distribution channels, we defined an indicator based on the difference in the number of institutions providing each drug (D = number of retail pharmacies – number of hospitals). When *D* > 1,000, a drug was classified as being “primarily supplied through retail pharmacies”; when *D* < −1,000, it was classified as “primarily supplied through hospitals.” Based on this criterion, 17 drugs were found to be concentrated mainly in designated retail pharmacies—primarily anti-inflammatory/antipyretic and antidiabetic drugs—while 28 drugs were concentrated in hospitals, including oncology therapies, cardiovascular emergency drugs, and perioperative medications (Supplementary Table S5).

### 3.2 Availability of drugs in key disease areas

Further analyses were conducted for three disease areas of national priority, which included a relatively large number of negotiated drugs: oncology (88 drugs), chronic diseases (40 drugs), and rare diseases (40 drugs).

#### 3.2.1 Oncology drugs

##### 3.2.1.1 Overall availability in healthcare institutions

Oncology drugs represent the largest therapeutic category among negotiated drugs, totaling 88 drugs. Given the specificity and severity of cancer, prior studies have confirmed that patients with cancer are more likely to seek treatment at higher-tier hospitals (3). Accordingly, this section focuses on the availability of oncology drugs in tertiary hospitals nationwide.

The average availability rate of oncology drugs in tertiary hospitals was 12.50%. Among oncology drugs newly negotiated in 2023 and 2024, 90.24% had an availability rate of less than 10% in tertiary hospitals (Supplementary Table S6). Such low availability suggests that many patients may face challenges in accessing the most appropriate and up-to-date therapies at their treating hospitals or within their local regions, thereby undermining long-term availability and convenience.

Only four oncology drugs exceeded a 50% availability rate in tertiary hospitals: camrelizumab for injection (65.12%), apatinib mesylate tablets (60.06%), pyrotinib maleate tablets (54.02%), and anlotinib hydrochloride capsules (50.78%). These drugs were introduced relatively early into the NRDL and target cancers with large patient populations, including non–small cell lung cancer (NSCLC), breast cancer, and lymphoma.

##### 3.2.1.2 Availability of drugs for high-incidence cancers

To further investigate the availability of oncology drugs for high-incidence cancers, we analyzed NSCLC, breast cancer, and lymphoma—three cancers with both high incidence and a relatively large number of negotiated drugs. The availability rates of these drugs in tertiary hospitals were 7.95, 16.21, and 14.91%, respectively.

Taking NSCLC as an example, a marked difference was observed between older and newly negotiated drugs in tertiary hospital availability (Table 4). Anlotinib hydrochloride capsules, which entered the NRDL in 2018, had an availability rate of 51% in tertiary hospitals, indicating that more than half of tertiary hospitals nationwide recognized the treatment demand of NSCLC patients. In contrast, most of the NSCLC drugs added in 2023 and 2024 had availability rates below 5%, reflecting persistently limited availability of newly negotiated drugs for patients.

**Table 4:** Availability of non–small cell lung cancer (NSCLC) drugs in tertiary hospitals.

Drug name	Negotiation year	Availability rate
Anlotinib hydrochloride capsules	2018	50.79%
Recombinant human endostatin injection	2017	26.81%
Alectinib hydrochloride capsules	2019	19.64%
Ensartinib hydrochloride capsules	2021	13.47%
Almonertinib mesilate tablets	2020	10.58%
Brigatinib tablets	2022	9.89%
Rilertinib mesylate tablets	2024	6.83%
Savolitinib tablets	2022	6.78%
Lorlatinib tablets	2022	6.39%
Befotertinib mesylate capsules	2023	5.38%
Iruplinalkib tablets	2023	4.59%
Furmonertinib mesilate tablets	2021	4.29%
Trametinib tablets	2020	3.60%
Entrectinib capsules	2023	3.36%
Paclitaxel polymeric micelles for injection	2024	2.76%
Unecritinib fumarate capsules	2024	2.27%
Envonalkib citrate capsules	2024	1.46%
Rezivertinib mesylate capsules	2024	1.09%
Sunvozertinib tablets	2024	0.86%
Capmatinib hydrochloride tablets	2024	0.69%
Tepotinib hydrochloride tablets	2024	0.49%
Repotrectinib capsules	2024	0.44%
Glumetinib tablets	2023	0.30%

#### 3.2.2 Drugs for chronic diseases

Among the sample, 40 drugs were indicated for the treatment of four major chronic diseases: hypertension, diabetes, hypercholesterolemia, and chronic obstructive pulmonary disease (COPD). National health policies have explicitly emphasized the principle of “receiving care close to home” in the management of chronic diseases. For example, the *Guiding Opinions on Promoting the Establishment of a Hierarchical Diagnosis and Treatment System* issued by the State Council in 2015 highlighted the separation of acute and chronic care. They advocated for “primary care as the first point of consultation,” encouraging the gradual transfer of stable chronic disease patients to primary-level facilities for management (26). More recently, the *Implementation Plan for Strengthening Primary Healthcare Services* (2025) further underscored the goal of enabling the public to seek care for minor illnesses, rehabilitation, and chronic disease management within their local communities (27). However, our findings indicate that tertiary hospitals remain the primary suppliers of drugs for the chronic diseases (Table 5).

**Table 5:** Average availability rates of chronic disease drugs in different levels of healthcare institutions and designated retail pharmacies.

Type of institution	Average availability rate (%)
Primary hospitals	0.39
Secondary hospitals	2.30
Tertiary hospitals	11.80
Retail pharmacies	0.27

From the perspective of designated retail pharmacies as a supplementary supply channel, the *Mid- to Long-Term Plan for the Prevention and Treatment of Chronic Diseases in China (2017–2025)* called for “leveraging the role of community pharmacies in ensuring the supply of drugs at the primary level to improve drug availability” (28). In our sample, 95% of the 40 drugs had been included in designated retail pharmacies. Nevertheless, only azilsartan ester tablets and dulaglutide injections had an availability rate above 1% in retail pharmacies, suggesting that while most chronic disease drugs have entered retail channels, their actual availability remains very limited.

#### 3.2.3 Drugs for rare diseases

Given the urgent clinical demand and high public attention surrounding rare diseases, the National Health Commission has introduced a series of initiatives to strengthen diagnosis and treatment capacity. These include the establishment of a National Rare Disease Diagnosis and Treatment Collaboration Network, comprising 419 hospitals with relatively strong capacities and higher caseloads of rare diseases. The network consists of one national leading hospital (Peking Union Medical College Hospital, Chinese Academy of Medical Sciences), 32 provincial leading hospitals, and 386 member hospitals, covering all 31 provinces in China. According to policy directives, these hospitals are expected to serve as the backbone for the diagnosis and treatment of rare diseases, incorporate rare disease drugs into formularies and essential supply lists, implement shortage warnings and reporting systems, and strive to meet clinical medication needs.

Results showed that, across the 40 rare disease drugs in the sample, the average availability rate was 33.79% in leading hospitals of the collaboration network and 15.30% across all network hospitals. Among the 33 provincial leading hospitals, the average number of rare disease drugs available was 13. However, 72.5% of rare disease drugs had an availability rate below 50% at these hospitals, with newly negotiated drugs exhibiting particularly poor availability. For example, in the 2024 negotiation batch, only daratumumab injection (subcutaneous) was available in more than 100 network hospitals, whereas deferasirox granules were available in only 9 hospitals, severely limiting patient access. Within the rare disease category, the ATC group of antineoplastic and immunomodulating agents—the largest subgroup in terms of the number of drugs—had an average availability rate of only 14.95% across all network hospitals (Supplementary Table S7).

Due to the scarcity of diagnostic and treatment resources for rare diseases, patients often have no choice but to seek care outside their home regions. In terms of geographic allocation, at the provincial level, only 7 of the sample drugs had entered collaboration network hospitals across all provinces, while 8 drugs were supplied in fewer than 50% of provinces. Even some rare disease drugs from earlier negotiation batches demonstrated poor availability, suggesting that although the establishment of the rare disease collaboration network has achieved certain progress, healthcare institutions remain inadequately supplied with rare disease drugs (Supplementary Table S7).

### 3.3 Provincial comparison of negotiated drugs allocation

The distribution of healthcare resources in China is uneven, and significant disparities exist in the allocation of negotiated drugs across provinces. This section compares the allocation of negotiated drugs across healthcare institutions and the retail pharmacies in all 31 provinces.

**Figure 4:**
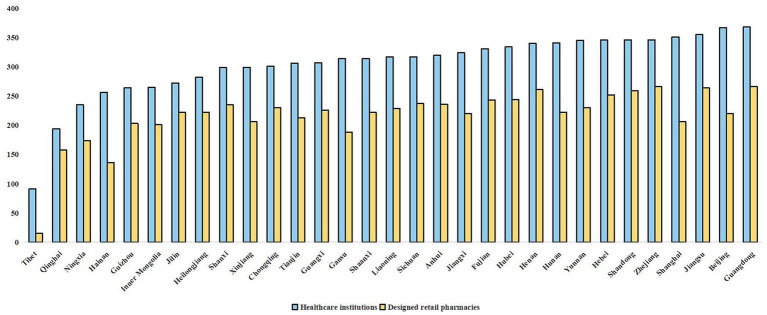
Number of negotiated drugs supplied by healthcare institutions and retail pharmacies across provinces.

At the national level, no province achieved full supply of all sample drugs. In terms of the number of drugs supplied by healthcare institutions, most provinces clustered between 299 and 368 (Figure 4), with an average of 304 drugs, accounting for 79% of the sample. The top five provinces were all in the more developed eastern region: Guangdong (368), Beijing (367), Jiangsu (355), Shanghai (351), Zhejiang (346), Shandong (346), and Hebei (346). By contrast, in designated retail pharmacies, the average number of drugs supplied was 216, with Tibet ranking lowest at only 15 (Figure 4).

**Figure 5:**
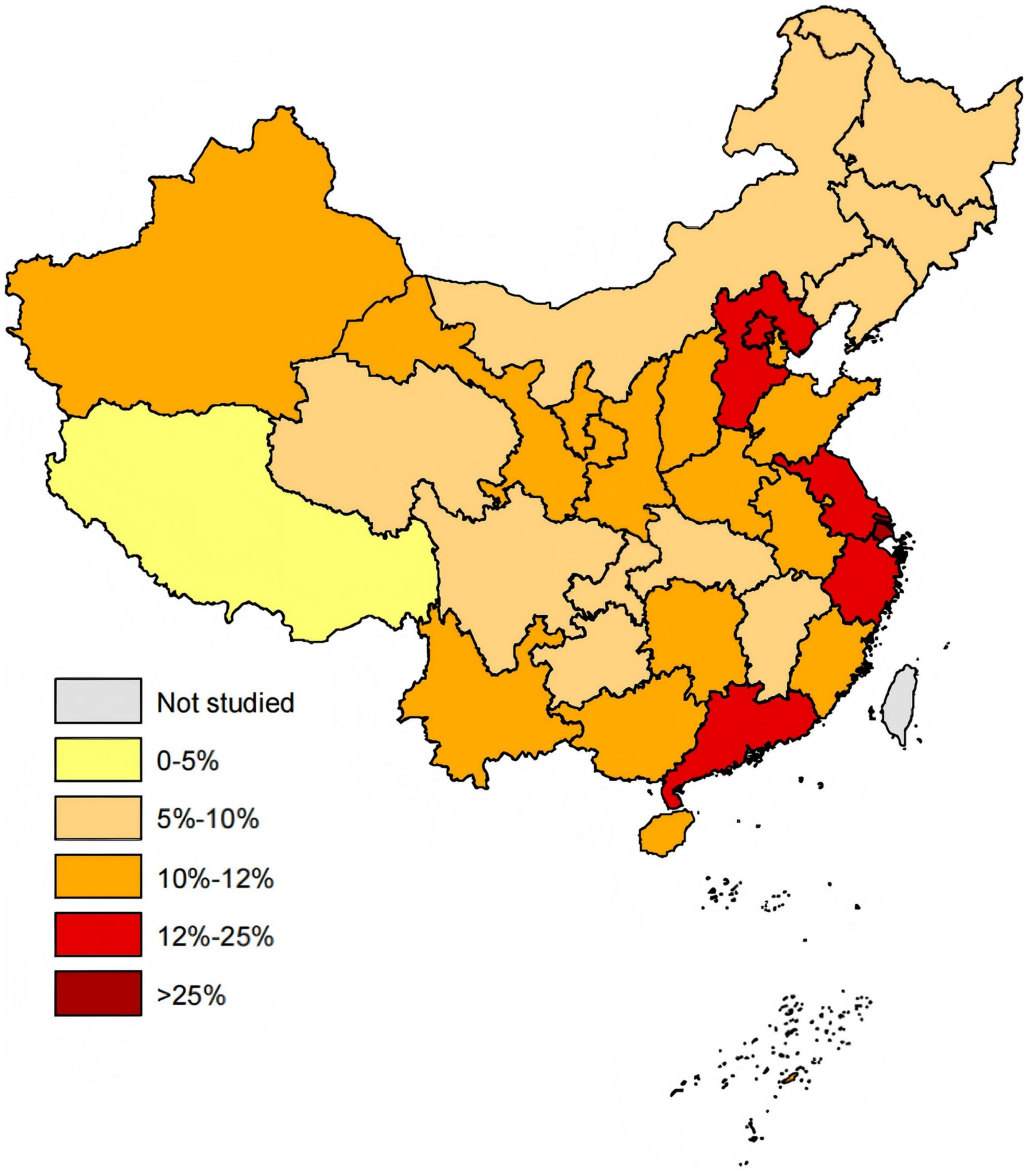
Average allocation rates of negotiated drugs in tertiary hospitals across provinces. Gray areas represent regions that have not been studied.

To further compare provincial differences in tertiary hospitals, the average allocation rate of drugs in tertiary hospitals was used as the key indicator. Results showed that the provincial averages largely fell within the range of 8–14%. Shanghai had the highest allocation rate at 26.83%, followed by Jiangsu at 14.29% (Figure 5). Zhejiang, Beijing, Hebei, Guangdong, and Fujian also demonstrated relatively high allocation rates. From a geographic perspective, provinces with higher allocation rates were predominantly located in the eastern and southern coastal regions, which are economically more developed and have greater concentrations of healthcare resources, revealing a clear “east–high, west–low” structural imbalance. Further calculations of the standard deviation (SD) and interquartile range (IQR) (see Appendix 1 for details) revealed that provinces with higher average drug allocation rates in tertiary hospitals also exhibited higher SD and IQR values, indicating greater differences in drug availability between healthcare institutions within these regions.

**Figure 6:**
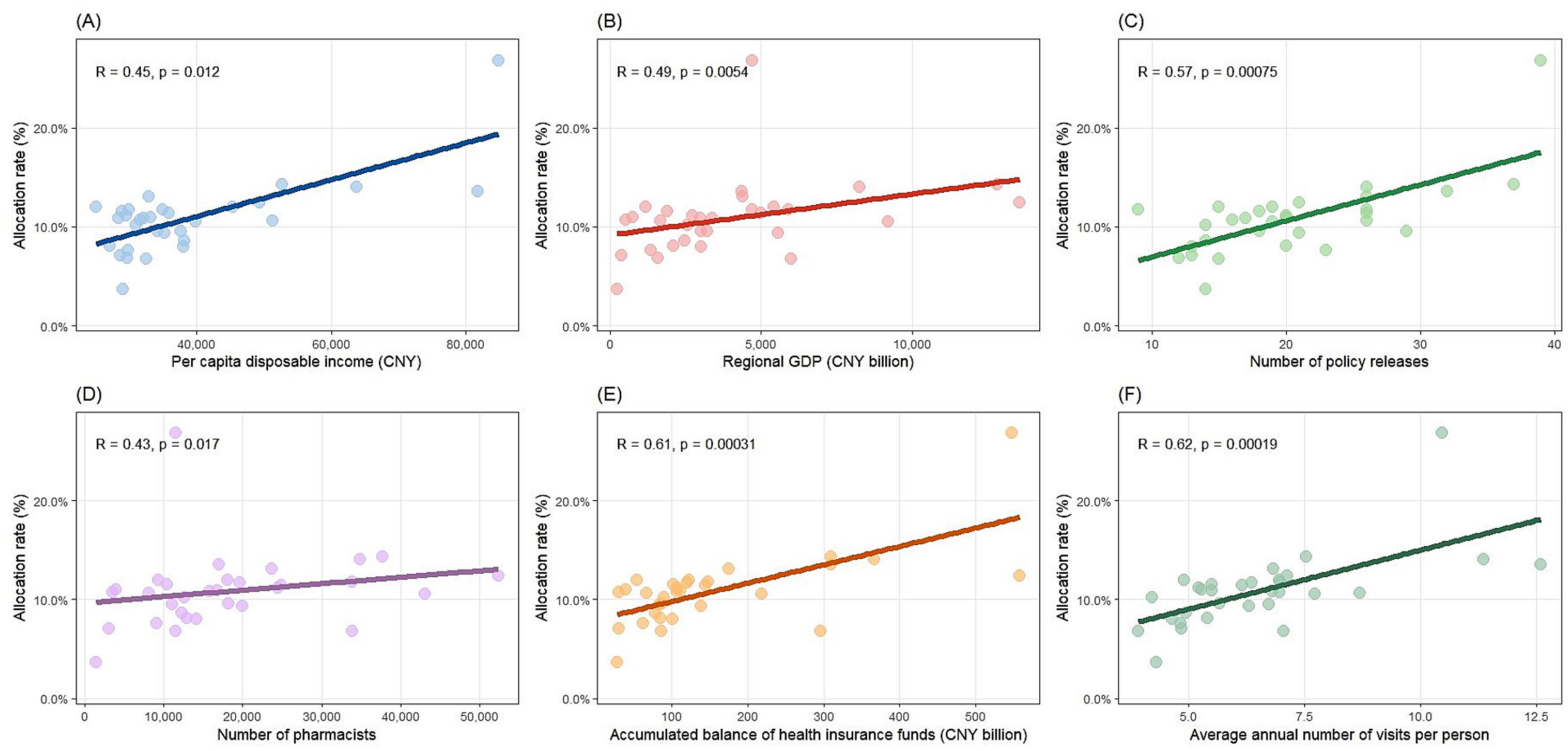
Correlation between provincial average allocation rates of negotiated drugs in tertiary hospitals and **(A)** *per capita* disposable income, **(B)** regional GDP, **(C)** number of policy releases, **(D)** number of pharmacists, **(E)** accumulated balance of health insurance funds, and **(F)** average annual number of visits per person.

Spearman’s rank correlation analysis further demonstrated significant positive associations between the average negotiated drug allocation rate in tertiary hospitals and several key variables, as shown in Figure 6. Specifically, there were significant positive correlations with: Per capita disposable income (*R* = 0.45, *p* = 0.012; Figure 6A), Regional GDP (*R* = 0.49, *p* = 0.005; Figure 6B), Number of relevant policy releases (*R* = 0.57, *p* < 0.001; Figure 6C), Number of pharmacists (*R* = 0.43, *p* = 0.017; Figure 6D), Accumulated balance of health insurance funds (*R* = 0.61, *p* < 0.001; Figure 6E), Average annual visits per person (*R* = 0.62, *p* < 0.001; Figure 6F). These results suggest that provinces with stronger economic capacity, more robust policy implementation, higher pharmaceutical management capacity, stronger healthcare security, and greater patient demand tend to have higher availability of negotiated drugs.

## 4 Discussion

This study provides a systematic analysis of the availability of negotiated drugs listed in the NRDL across China, covering a wide range of therapeutic categories and provinces. The findings reveal that the overall availability of negotiated drugs in healthcare institutions remains low and unevenly distributed. Availability varied by type of institution, healthcare tier, drug categories, and formulation. Moreover, the timing of formulary inclusion, regional economic status, the number of policy releases, the number of pharmacists, the accumulated balance of health insurance funds, and the average annual number of visits were all shown to influence availability.

### 4.1 Hospital-level availability analysis

The level of healthcare institutions reflects their overall capacity, including scale, medical equipment, and available technologies. Research has shown that the availability of negotiated drugs in tertiary hospitals is significantly higher than that in secondary hospitals, primary healthcare institutions, and retail pharmacies. This aligns with their roles in subject development, specialized treatment capabilities, and policy orientation (3). According to policy requirements, tertiary hospitals are primarily responsible for managing critical, severe, and complex diseases, making them more likely to be the first to supply negotiated drugs (29). Additionally, critically ill patients tend to seek care in tertiary hospitals (30), further increasing the availability of negotiated drugs in these institutions. Despite their relative advantage, the overall availability of negotiated drugs in tertiary hospitals remained low at 9.84%. Negotiated drugs, often high-value and innovative, demand stringent management, infrastructure, logistics, and cold chain systems. These factors impose dual constraints, both financial and technical, on secondary and lower-level institutions. Under current conditions, therefore, prioritizing the improvement of negotiated drug availability in tertiary hospitals appears to be the most feasible approach, offering higher marginal benefits.

#### 4.1.1 Analysis of availability in key disease areas

High-priority drugs targeting cancer and chronic diseases, availability averaged just around 12%. For chronic disease drugs, however, achieving the policy goal of “receiving care close to home” will require a stratified strategy: while innovative drugs should be prioritized in tertiary hospitals, commonly used chronic disease treatments that are clearly diagnosed, stable, and well controlled should be gradually extended to secondary and primary facilities as well as retail pharmacies, ensuring both innovation at higher tiers and adequate supply at the grassroots level.

The case of oncology drugs illustrates this dynamic. Four widely used oncology drugs—camrelizumab injection (65.12%), apatinib mesylate tablets (60.06%), pyrotinib maleate tablets (54.02%), and anlotinib hydrochloride capsules (50.78%, 2020, non–small cell lung cancer)—all had availability rates above 50% in tertiary hospitals, markedly higher than most other oncology drugs on the NRDL. This suggests that high-incidence cancers with large patient populations drive hospitals’ willingness to supply such drugs, underscoring the influence of disease epidemiology on availability (31, 32).

Furthermore, the relatively high availability of these drugs is closely linked to their established clinical value. Camrelizumab, one of the first PD-1 inhibitors launched domestically, has demonstrated substantial efficacy and safety in lymphoma and non–small cell lung cancer (33). Apatinib, the first VEGFR-2 inhibitor approved in China, achieved an objective response rate of 43.2% in advanced gastric cancer, significantly higher than traditional chemotherapy (34). Pyrotinib, the first domestically developed irreversible dual HER2 inhibitor, has effectively suppressed HER2 mutations in breast cancer, significantly prolonging progression-free survival (35). Anlotinib, the first domestically developed multi-target TKI approved for third-line NSCLC treatment, has been shown to significantly improve both overall survival and quality of life (36). Their higher availability thus reflects not only their innovative mechanisms of action but also their strong clinical evidence base and therapeutic value. As accumulating evidence has positioned these drugs as standard-of-care treatments, they have been highly recommended by national and professional clinical guidelines (37). This robust clinical endorsement has facilitated their broader adoption in tertiary hospitals. These findings highlight the need to revisit the fundamental purpose of drug price negotiations: ensuring meaningful patient benefit. The inclusion of innovative drugs in the NRDL should not be driven solely by price reductions. Still, it must prioritize “true innovation”—not only in terms of novel mechanisms of action but also in demonstrable clinical benefits and safety. Only when drugs significantly improve survival, enhance quality of life, and strengthen adherence can reimbursement decisions be said to reflect “value-based purchasing.” Such an approach would ensure that negotiated drugs are not only listed in the formulary but are also widely adopted and effectively used in clinical practice.

Although China has established a Rare Disease Diagnosis and Treatment Collaboration Network to improve access, current data indicate that availability remains limited. Among the 40 rare disease drugs examined, the average availability rates in leading hospitals and all member hospitals of the collaboration network were 33.79 and 15.30%, respectively, with only seven drugs reaching hospitals in all provinces. Policy and financial support mechanisms should therefore prioritize the introduction and rational use of rare disease drugs with high clinical value in collaboration with hospitals. Their provision and use should be incorporated into performance evaluation systems to create effective incentives. Moreover, leading hospitals within the network should serve as exemplars by promoting multicenter drug evaluations and evidence-based dissemination, supported through standardized treatment pathways, clinical training, and academic exchange, thereby strengthening the overall level of rare disease management.

#### 4.1.2 Analysis of differences in availability between traditional Chinese and Western drugs

The analysis of the availability of Western and TCM revealed that the availability of Chinese patent medicines is generally lower. This disparity may stem from several factors. First, the majority of physicians, trained predominantly in Western medicine, lack expertise in the diagnostic and therapeutic practices of TCM. This lack of knowledge makes the rational use of Chinese patent medicines difficult to ensure, thereby hindering their clinical promotion. Second, healthcare cost-control policies have, to some extent, restricted the use of TCM, especially those treatments categorized as adjunctive therapies within insurance allocation. In contrast, Western medicines, with their well-established efficacy, strong support from clinical guidelines, and favorable policy incentives, tend to have more opportunities for use. Furthermore, compared to Western medicines, TCM has relatively limited evidence within modern medical systems and lacks international recognition. This results in lower awareness and acceptance of TCM in clinical practice, which contributes to its significantly lower availability compared to Western medicines. Thus, the differences in availability between Western and Chinese patent medicines are not solely attributable to the characteristics of the drugs themselves, but are also influenced by deeper factors such as physicians’ educational backgrounds, healthcare policies, and clinical guidelines.

#### 4.1.3 The impact of drug inclusion timing on availability

The timing of inclusion in the NRDL is a key factor influencing drug availability. Studies have shown that drugs included earlier in the NRDL generally have higher availability compared to those added more recently. Among the factors affecting the availability of negotiated drugs, the timing of government reimbursement negotiations is an important facilitating factor. The longer a drug has been included in the NRDL, the higher the likelihood of its availability in healthcare institutions. This lower availability reflects a time lag between adjustments to the medical insurance catalog and actual hospital supply (38). Specifically, for newly added drugs, hospitals tend to adopt a cautious stance regarding their clinical efficacy, safety, and cost-effectiveness evidence. As a result, initial allocation rates may be relatively low. Additionally, this phenomenon may also stem from delays in administrative processes and approvals. Once a drug is included in the NRDL, healthcare institutions typically need to undergo processes such as formulary review and approval by the pharmacy and therapeutics committee, which can take several months, creating a structural lag (39, 40).

Although the waiting period for innovative drugs to enter the NRDL has shortened dramatically—from nearly 5 years in the past to less than 2 years on average, with some drugs included within 6 months of approval (41)—concerns remain. Many of these drugs are initially approved based on limited evidence, such as small samples, single-arm trials, or surrogate endpoints, leaving their real-world effectiveness and safety uncertain (42). Premature inclusion and rapid diffusion may therefore lead to uncertain clinical benefits, heightened patient safety risks, and challenges to the sustainability of insurance funds (43). In cases where effective alternatives already exist, a moderate delay in formulary inclusion and uptake may be beneficial. Such an approach would allow more time for real-world evidence to accumulate, thereby balancing insurance fund sustainability with public health interests, while ensuring patients continue to receive safe and effective treatments.

#### 4.1.4 National negotiated drugs allocation strategies

While current policies promote the supply of negotiated drugs, requiring all hospitals to supply every NRDL drug is impractical. It is therefore essential to define a reasonable scope of “essentially required” drugs, implement differentiated provision requirements, and strengthen hospitals’ motivation to comply. Externally, performance evaluation systems should be adjusted to remove conflicting indicators, such as the essential drugs ratio, which may discourage the use of negotiated drugs. Positive incentive lists could also be introduced, incorporating indicators such as rational use and patient benefit rates, with preferential insurance funding and recognition given to high-performing institutions.

Internally, hospitals must recognize that evaluation indicators are designed to prioritize patient health rather than simply maximize the number of listed drugs. Institutions should leverage pharmacists’ expertise to comprehensively assess negotiated drugs against existing formulary, centralized procurement, and clinical demand, while considering efficacy, cost, and insurance restrictions. Hospitals should then communicate evidence-based requests to health authorities and insurance agencies, ensuring that urgently needed, highly effective drugs are included in hospital formularies. This would enhance insurance fund efficiency, reduce unnecessary spending, and ensure that patients have access to optimal treatment.

### 4.2 Pharmacy-level availability analysis

Retail pharmacies primarily supplied anti-inflammatory/antipyretic and antidiabetic drugs, with oral and topical formulations more widely available. These drugs align with patients’ needs for self-management, explaining their concentration in retail outlets. By contrast, injectable and inhalation formulations faced greater challenges due to specialized administration requirements, storage conditions, and reliance on professional supervision. Previous studies have noted that many hospitals refuse to administer injectable drugs obtained outside their own pharmacies due to concerns over quality control and patient safety (44, 45). Consequently, patients face practical barriers when obtaining drugs from retail pharmacies, limiting the broader distribution of such drugs. This suggests that pharmacies emphasize availability and convenience, whereas hospitals remain critical for drugs requiring clinical oversight. To expand pharmacy-based access, a joint regulatory mechanism involving the National Healthcare Security Administration and the National Health Commission could be established to ensure quality control throughout the prescription-to-dispensation process, reduce hospitals’ concerns about extra-institutional drugs, and encourage wider provision of negotiated drugs in pharmacies.

Among drugs not yet supplied by pharmacies, 64.52% were newly added in 2023 and 2024, suggesting slower uptake in retail channels. Compared to hospitals, pharmacies face additional hurdles, such as establishing dedicated counters, hiring licensed pharmacists, upgrading insurance billing systems, and meeting storage and distribution requirements. At the same time, the zero-markup policy reduces profit margins, while other policies—such as integration into outpatient insurance pooling and centralized procurement—exert further downward pressure on retail pricing (46, 47). These factors combine to constrain pharmacies’ financial viability. To address this, the NHSA could consider allowing modest markups on certain high-cost drugs, particularly those with significant storage and transportation expenses. However, robust oversight is essential to prevent insurance fraud and ensure transparent and efficient use of funds.

### 4.3 Provincial-level drug availability disparity analysis

Significant disparities in the availability of negotiated drugs are observed across the 31 provinces, with factors such as regional GDP, per capita disposable income, and the number of policy releases significantly influencing drug availability. Consistent with previous research indicating that critical care and healthcare services are often concentrated in economically developed regions (48–50), our findings demonstrate that provinces with higher allocation rates are primarily located in China’s economically developed central and eastern regions, such as Guangdong, Beijing, Jiangsu, and Shanghai. These regions typically benefit from a stronger economic foundation, higher disposable incomes, more proactive policymaking, and a higher concentration of healthcare resources and pharmaceutical enterprises, which facilitates the development of more robust drug supply chains. Further analysis of the SD and IQR reveals that provinces with higher drug allocation rates in tertiary hospitals also exhibit higher SD and IQR values. This phenomenon may be due to better overall drug provision in these regions, with a stronger emphasis on tailoring drug distribution to the specific characteristics of different hospitals, particularly in terms of hospital specialization and patient demographics. However, it may also reflect an imbalance in the internal allocation of drug supplies, suggesting that some hospitals face challenges related to insufficient drug availability or poor management practices. To address this issue, we recommend that policymakers and relevant authorities place greater focus on ensuring more balanced drug distribution within regions to reduce internal disparities in drug provision.

From a policy and fiscal perspective, the eastern regions have long benefited from a more developed fiscal decentralization and resource allocation mechanism. Since the tax-sharing reform, the expansion of local fiscal autonomy has allowed economically advanced regions to more flexibly utilize local health insurance surpluses, fiscal subsidies, and policy innovation pilot programs to support the procurement and reimbursement of negotiated drugs (51). Additionally, these regions have accumulated more experience in policy implementation and possess higher levels of informatization, which has expedited the execution of drug access and reimbursement policies. In contrast, central and western regions face challenges such as insufficient fiscal revenue and significant pressure on health insurance funds, resulting in delays in the drug supply process. Therefore, we believe that increasing policy support in these regions is crucial. By improving the availability and affordability of negotiated drugs in healthcare institutions in these areas, it will be possible to promote more balanced development of the healthcare system and enhance the overall efficiency of health insurance resource utilization.

### 4.4 Policy implications for other countries

For other middle-income countries, these findings suggest that merely relying on price negotiations to include new drugs in the reimbursement list does not automatically translate into real clinical availability. In order for patients to truly benefit from these drugs, policies must simultaneously address three key implementation-level questions: which institutions should prioritize providing these drugs, whether these institutions have the motivation to supply them, and whether this provision can be sustained within the constraints of medical insurance funds. Based on the practical challenges revealed during the implementation of negotiated drugs in China, three policy directions that may offer valuable lessons are proposed: First, clearly define the scope of “must-supply” drugs, rather than requiring all healthcare institutions to supply all negotiated drugs. Instead, high clinical-value drugs with significant clinical demand should be prioritized for institutions with the necessary clinical capacity. Second, avoid creating rigid conflicts between cost-control indicators and new drug usage in performance assessments, preventing hospitals from passively restricting new drug use due to concerns about evaluation risks. Third, establish positive incentives by linking patient benefits, appropriate usage levels, and other relevant metrics to fiscal support or resource allocation, thus turning the issue of “hesitancy to supply” into “willing to supply and supply appropriately.” For other middle-income countries, when rolling out newly added drugs to the reimbursement list, considering these three aspects in advance will help turn “listing in the reimbursement list” into “true availability” while controlling the sustainability risks of medical insurance funds.

### 4.5 Limitations and directions for future research

This study has several limitations. First, the primary data were self-reported by pharmaceutical companies, which may introduce inconsistencies or reporting biases due to differences in reporting criteria or statistical errors. Second, the cross-sectional design captures only a snapshot of the current situation, while drug availability is a dynamic process that changes over time. Future research should incorporate longitudinal data and continuous tracking to improve the comprehensiveness and reliability of the findings. Furthermore, while this study considered several factors influencing drug availability, the scope remains limited. Future studies could expand the analytical framework by including qualitative interviews and additional variables, offering a more holistic understanding of the factors shaping negotiated drug availability in Chinese healthcare institutions. Additionally, the affordability of drugs, especially for low-income groups and rural populations, remains a critical issue. Future research could explore the relationship between drug prices, insurance allocation, and patient economic burdens, with a particular focus on the affordability of negotiated drugs for rural residents and low-income groups, to ensure that patients can access necessary treatments while minimizing their economic burden.

## Data Availability

Publicly available datasets were analyzed in this study. This data can be found: https://fuwu.nhsa.gov.cn/nationalHallSt/#/search/allocation-mechanism-search.

## Appendix

**Table A1:** Standard deviation (SD) and interquartile range (IQR) of the average allocation rate in tertiary hospitals across provinces.

Province	SD	IQR
Anhui	6.88030%	8.74636%
Beijing	11.92516%	13.62974%
Fujian	10.36467%	14.28571%
Gansu	12.36754%	12.68222%
Guangdong	11.39514%	13.11953%
Guangxi	9.54332%	13.26531%
Guizhou	6.80726%	7.72595%
Hainan	10.97673%	14.28571%
Hebei	10.44431%	15.74344%
Henan	10.06771%	11.66181%
Heilongjiang	6.79052%	9.32945%
Hubei	9.78521%	10.86006%
Hunan	11.07480%	11.29738%
Jilin	7.37760%	9.76676%
Jiangsu	10.44701%	13.70262%
Jiangxi	10.10346%	10.86006%
Liaoning	7.64177%	10.56851%
Inner Mongolia	7.81818%	12.31778%
Ningxia	11.04784%	10.49563%
Qinghai	8.60262%	7.28863%
Shandong	8.30762%	11.22449%
Shanxi	8.19981%	11.80758%
Shaanxi	9.21972%	14.21283%
Shanghai	17.83736%	22.88630%
Sichuan	5.70835%	6.99708%
Tianjin	7.56775%	10.49563%
Tibet	3.47248%	2.98834%
Xinjiang	10.29638%	12.53644%
Yunnan	12.23041%	12.39067%
Zhejiang	9.18333%	14.28571%
Chongqing	7.89043%	12.46356%
